# Odor as a Symptom of Disease

**Published:** 1896-07

**Authors:** 


### SELECTIONS.

### Odor as a Symptom of Disease.

The chief functions of the nose are: (1) Respiratory; (2) olfaction; (3) resonator to the voice; (4) office of regulator to the aeration of the middle ears.

The normal daily secretion of the nose is about one pint, which comes chiefly from the turbinated bodies, and is used in moistening the air before it reaches the lower respiratory organs. The nasal chambers heat the air for respiration, and aid in modulating and modifying voice sound, giving it proper resonance. Infinitesimal odorous particles dissolved and floating in the air are carried into the nasal fossæ and impinge upon the hairy terminations of the nerve filaments; thence the sensation is conveyed to the olfactory centers. There is but little loss in weight of musk and other strongly odorous substances after they have freely evolved their effluvia for several years. It is the mucous-membrane of the upper half of the nasal fossæ that is capable of appreciating odorous impressions.

Like the other special senses, olfaction may be cultivated by attention and practice. Experts can discriminate qualities of wines, liquors, drugs, etc.

Diseases have their characteristic odors. Persons who have visited many different insane asylums recognize the same familiar odor of the insane. *General paralysis* of the insane affords us a typical example. It is a true cerebral disease, physiologically, pathologically, and psychologically. In it the substance of the convolutions of the brain—the highest in quality and function of any organic product yet known in nature—undergoes a process of degeneration or atrophy, which finally invades the whole nervous system. The nerve and mind tissue die slowly and progressively. The blood-current carries the waste tissue to the lungs for aeration, and the result is the foul characteristic odor of this disease.

It is not insane asylums alone, but prisons, jails, workhouses, armies in camp, churches, schools, and nearly every household, that have their characteristic odors. It is when the insane, the prisoners, and the soldiers are aggregated in large groups or battalions that their characteristic odor is recognized by our much-neglected "smeller."

Most diseases have characteristic odors, and by the exercise of the sense of smell they could be utilized in differential diagnosis. For example: Favus has a mousey odor; rheumatism has a copious, sour-smelling acid sweat. A person afflicted with pyæmia has a sweet, nauseating breath. The rank, unbearable odor of pus from the middle ear tells the tale of the decay of osseous tissue. In scurvy the odor is putrid; in chronic peritonitis, musky; in syphilis, sweet; in scrofula, like stale beer; in intermittent fever, like fresh-baked brown bread; in fevers, ammoniacal; in hysteria, like violets or pineapple. Measles, diphtheria, typhoid fever, epilepsy, phthisis, etc., have characteristic odors.

The acuteness of the sense of smell is far greater in many of the lower animals (dogs, for example) than in man, and they employ it in guiding them to their food, in warning them of approaching danger, and for other purposes. The sphere of the susceptibility to various odors is more uniform and extended in man. The sense of smell is capable of great cultivation. For example: In the well-known case of James Mitchell, who was deaf and blind from birth, the sense of smell was his principal means of distinguishing persons and perceiving the approach of strangers.

Among many savage tribes the sense of smell is almost as acute as in many of the lower animals. Humboldt says the Peruvian Indians are able, in the middle of the night, to distinguish whether an approaching stranger is a European, American, Indian, or negro.—*Dr. J. H. M'Cassey, of Dayton, O., in Cincinnati Lancet-Clinic, June 6th.*

